# The Complete Plastome Sequence of an Antarctic Bryophyte *Sanionia uncinata* (Hedw.) Loeske

**DOI:** 10.3390/ijms19030709

**Published:** 2018-03-01

**Authors:** Mira Park, Hyun Park, Hyoungseok Lee, Byeong-ha Lee, Jungeun Lee

**Affiliations:** 1 Unit of Polar Genomics, Korea Polar Research Institute, Incheon 21990, Korea; mira0295@kopri.re.kr (M.P.); hpark@kopri.re.kr (H.P.); 2 Department of Life Science, Sogang University, Seoul 04107, Korea; 3 Polar Science, University of Science & Technology, Daejeon 34113, Korea

**Keywords:** *Sanionia uncinata*, chloroplast genome, Antarctic bryophyte, moss, plastome

## Abstract

Organellar genomes of bryophytes are poorly represented with chloroplast genomes of only four mosses, four liverworts and two hornworts having been sequenced and annotated. Moreover, while Antarctic vegetation is dominated by the bryophytes, there are few reports on the plastid genomes for the Antarctic bryophytes. *Sanionia uncinata* (Hedw.) Loeske is one of the most dominant moss species in the maritime Antarctic. It has been researched as an important marker for ecological studies and as an extremophile plant for studies on stress tolerance. Here, we report the complete plastome sequence of *S*. *uncinata*, which can be exploited in comparative studies to identify the lineage-specific divergence across different species. The complete plastome of *S*. *uncinata* is 124,374 bp in length with a typical quadripartite structure of 114 unique genes including 82 unique protein-coding genes, 37 tRNA genes and four rRNA genes. However, two genes encoding the *α* subunit of RNA polymerase (*rpoA*) and encoding the cytochrome b_6/f_ complex subunit VIII (*petN*) were absent. We could identify nuclear genes homologous to those genes, which suggests that *rpoA* and *petN* might have been relocated from the chloroplast genome to the nuclear genome.

## 1. Introduction

Antarctic terrestrial ecosystems are dominated by lichens and bryophytes (including mosses, liverworts and hornworts), encompassing more than 200 lichens and 109 mosses species [1]. Only two vascular plant species have survived and adapted to these extreme environments, with a very limited distribution restricted to the maritime Antarctic, while mosses are common plants on extensive ice-free areas of Antarctica.

*Sanionia uncinata* is one of the most dominant moss species in Antarctica and is mainly distributed over coastal areas [2,3]. Moreover, *S*. *uncinata* is distributed across multiple geographic regions, ranging from Northern Hemisphere (Europe, Asia, North America and the Arctic) to Southern Hemisphere (Africa, South America and the Antarctica) and also found at high-altitude mountains in tropical and subtropical areas [4]. A recent phylogeographic study has established the haplotype networks of *S*. *uncinata* populations by identifying their genetic diversity with massive molecular marker datasets of more than 200 specimens collected from various regions around the world [4].

*S*. *uncinata* is a pleurocarpous moss species that form dense and extensive carpets on terrestrial habitats over a wide range of water regimes, from dry rock surfaces to wet areas at the edges of streams or melt pools [5,6,7]. *S*. *uncinata* has been extensively used as an experimental model for the study of environmental impacts on plants [8,9,10]. This species is known to tolerate dehydration by retaining moisture in their tissues for a long period of time by forming a carpet-like community shape that helps to avoid water loss [11]. However, the molecular mechanism and molecular ecology underlying stress tolerance have yet to be elucidated*.* The dehydration process, although it prevents the moss from freezing, directly affects cell metabolism and as a result, photosynthetic capacity decreases when the water content of moss drops below the optimum level [12]. On the other hand, photosynthesis is essential for the production of energy needed for plant growth and takes place in the chloroplasts. Thus, the integrity and metabolic performance of chloroplasts are very important for photosynthetic activities [13].

Chloroplasts are unique organelles, derived from cyanobacteria through endosymbiosis, that provide essential energy for plants and algae through photosynthesis [14,15]. They contain their own genomes that have a unique mechanism of RNA transcription and are inherited maternally. Chloroplasts are known to play an important role in the synthesis of pigments, starch, fatty acids and amino acids as well as the photosynthesis process [16,17]. In general, chloroplast genomes—namely, plastomes—are highly conserved with regards to gene sequences and gene content in terrestrial plants. Their highly conservative nature is sufficient to perform comparative studies on different species to discuss evolutionary relationships between species in terms of molecular phylogeny and molecular ecology [18]. For instance, plastome sequences provide species-specific information that includes genome size, gene order, genome rearrangements, patterns of base pair composition, codon usage, massive plastid gene losses and various type of nucleotide polymorphism [19,20].

Despite their genetic diversity and evolutionary significance, genetic resources for bryophytes are very limited when compared to angiosperms. Of the 2352 records for chloroplast genome sequences of green plants, only 15 plastomes of bryophytes comprised of 2 from hornworts, 5 from liverworts and 8 from mosses (including *Sanonia uncinata* NC_025668 which was directly submitted by the authors of this study) are available in public repositories [21] (http://www.ncbi.nlm.nih.gov). There are currently only four complete chloroplast genomes fully published for mosses, for example, *Physcomitrella patens* [22], *Tortula ruralis* [13], *Tetraphis pellucida* [23] and *Tetraplodon fuegianus* [24]. The plastome information of more bryophytes and comparative genomic studies are necessary to better understand the molecular evolutionary events or functions of chloroplast genes. In this regard, the plastome information of *S*. *uncinata* provided in this study will be a very useful resource for future research on the ecology, physiology and molecular evolution of bryophytes.

## 2. Results and Discussion

### 2.1. Overall Genome Organization

Illumina MiSeq sequencing produced 4,993,466 raw reads with an average read length of 301 bp and a total number of 1,503,033,266 base pairs. A total of 46,573 chloroplast-related reads were obtained as a result of alignment of quality trimmed reads against other chloroplast genomes publically available in NCBI. Assembly of the nucleotide sequence reads was performed to obtain non-redundant contigs and singletons using CLC Genomics Workbench V7.5 (CLC bio, Aarhus, Denmark). The final *S*. *uncinata* plastome sequence has been submitted to GenBank (Accession: NC_025668).

**Figure 1:**
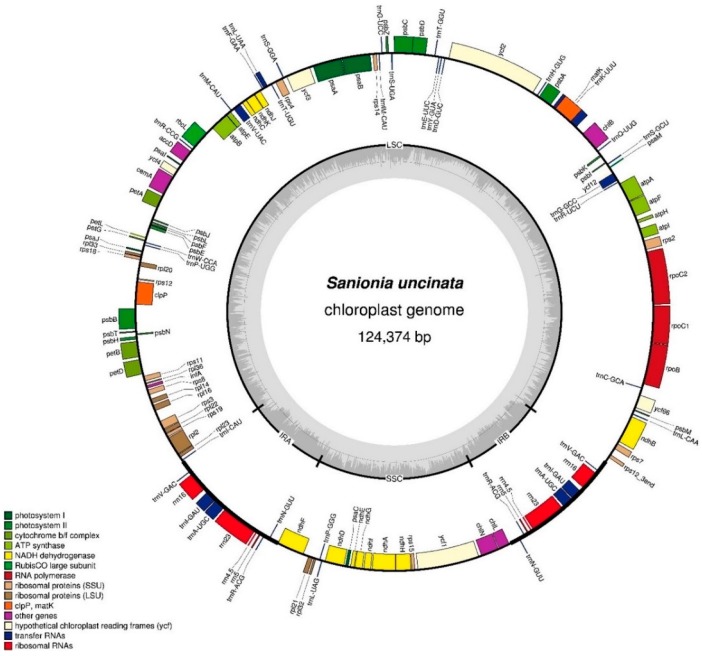
Map of the *Sanionia uncinata* plastome. Complete plastome sequences were obtained from the de novo assembly of Illumina paired-end reads. Genes are color coded by functional group, which are located in the left box. The inner darker gray circle indicates the GC content while the lighter gray corresponds to AT content. IR, inverted repeat; LSC, large single copy region; SSC, small single copy region. Genes shown on the outside of the outer circle are transcribed clockwise and those on the inside counter clockwise. The map was made with OGDraw [31].

The gene map for the *S*. *uncinata* plastome is shown in Figure 1. The complete plastome of *S*. *uncinata* is 124,374 base pairs (bp) in length with a typical quadripartite structure including large and small single-copy regions (LSC of 86,570 bp and SSC of 18,430 bp) separated by a pair of identical inverted repeats (IRA and IRB) of 9687 bp each (Figure 1). Most of the chloroplast DNA had a well preserved quadripartite structure in bryophytes [23,25,26] and vascular plants [27,28]. The genome contained 114 unique genes including 82 unique protein-coding genes, 37 tRNA genes and 4 rRNA genes (Table 1). The gene content of the IR regions was conserved among *S*. *uncinata, T*. *ruralis* and *P*. *patens.*

The size of the plastome of *S*. *uncinata* is very similar to those of liverworts (*Marchantia polymorpha* 121,024 bp NC_001319, *Pellia endiviifolia* 120,546 bp NC_019628, *Aneura mirabilis* 108,007 bp NC_010359, *Ptilidium pulcherrimum* 119,007 bp NC_015402) and mosses (*Physcomitrella patens* 122,890 bp NC_005087, *Tortula ruralis* 124,374 bp NC_012052, *Tetraphis pellucida* 127,489 bp NC_024291) and *Tetraplodon fuegianus* 123,670 bp, KU_095851(unverified) but much smaller than hornworts (*Anthoceros formosae* 161,162 bp NC_004543 and *Nothocerosaenigmaticus* 153,208 bp NC_020259), which have an increased length of intragenic spacers in the LSC region or in the identical IR regions [18,29,30].

The overall G/C content was 29.3% for *S*. *uncinata*, similar to other known bryophyte plastomes (*P*. *patens* (28.5%) [22], *T*. *pellucida* (29.4%) [23], *T*. *fuegianus* (28.7%) [24]), as well as the liverwort *M. polymorpha* (28.8%) [26], hornwort *A. formosae* (32.9%) [25], the charophyte *Chaetosphaeridium* (29.6%) [32] and algae (30–33%) [22] but significantly less than the 34~40% found in seed plants [33].

The chloroplast genes found in the complete plastome are represented in Table 2. There were 14 intron-containing genes including 5 tRNA genes and 9 protein-coding genes and almost all of which were single-intron genes except for *ycf3* and *clpP*, which each had two introns. Two exons of trans-spliced gene *rps12* are located in the LSC 71 kb apart from each other. The *trnK-UUU* gene has the largest intron (2272 bp), which has *matK* ORF (1548 bp) encoding a maturase involved in splicing type II introns [34].

The 82 protein-coding genes in this genome represented nucleotide coding for 40,330 codons. On the basis of the sequences of protein-coding genes and tRNA genes within the plastome, the frequency of codon usage was deduced (Table 3). Among these codons, 4343 (10.85%) encoded for leucine and 455 (1.14%) for Tryptophan, which were the most and the least amino acids, respectively. The codon usage was biased towards a high representation at the third codon position. A biased frequency of codons included the levels of available tRNA, functionally related genes, evolutionary pressures and the rate of gene evolution [35].

### 2.2. Comparison with Other Bryophyte Plastomes

**Figure 2:**
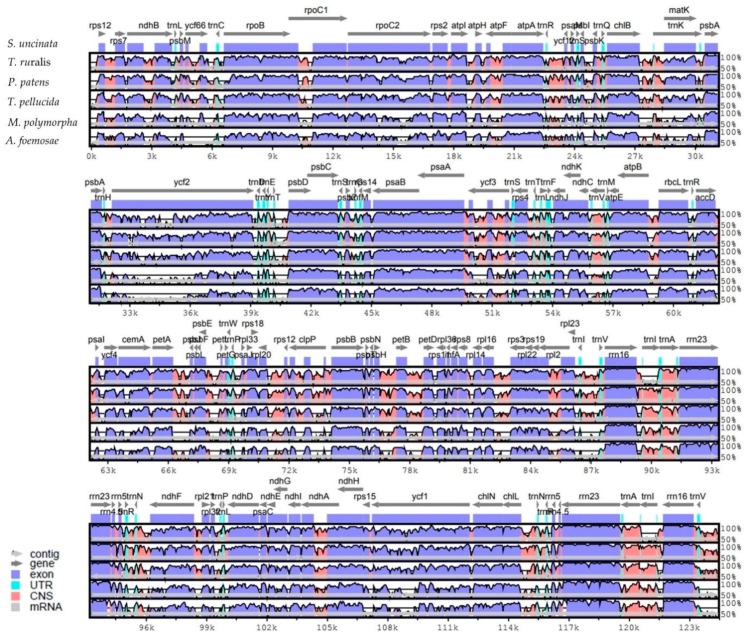
Alignment of complete plastome sequences from six species. Alignment and comparison were performed using mVISTA and the percentage of identity between the plastomes was visualized in the form of an mVISTA plot. The sequence similarity of the aligned regions between *S. uncinata* and other five species is shown as horizontal bars indicating average percent identity between 50–100% (shown on the y-axis of graph). The x-axis represents the coordinate in the plastome. Genome regions are color-coded for protein-coding (exon), rRNA, tRNA and conserved non-coding sequences (CNS) as the guide at the bottom-left.

Multiple complete bryophyte plastomes available provide an opportunity to compare the sequence variation at the genome-level. We therefore compared the whole plastome sequence of *S. uncinata* with those of mosses *T. ruralis*, *P. patens*, *T. pellucida*, the liverwort *M. polymorpha* and hornwort *A. formosae*. The sequence identity between all five bryophyte plastomes was plotted using the mVISTA program with the annotation of *S. uncinata* as a reference (Figure 2).

Sequence similarities of the genes between *S. uncinata* and other bryophytes (mosses, liverwort and hornwort) were compared (Table S1). The *rRNA* genes (*rrn5*, *rrn16*, *rrn4.5* and *rrn23*) in the IRs region showed the highest sequence similarity (average 98.3–96.1%) and PSII-associated genes such as *psbL*, *psbA*, *psbN*, *psbZ*, *psbE*, *psbH*, *psbK*, *psbF*, *psbB*, *psbD* and *psbJ*, which also displayed high levels of sequence similarity (average 93.9–89.8%). Genes for large subunit ribosomal proteins (*rpl32*, *rpl20*, *rpl33* and *rpl23*) and a small subunit ribosomal protein (*rps12*) were relatively more conserved than other coding genes. Notably, the highest sequence variation occurred in the *matK* gene (average 79.4%), widely known to be evolved rapidly and thus often used as a barcoding marker in phylogenetic and evolutionary studies [34,36], suggesting that this gene has also undergone evolutionary pressure within bryophytes. Following this, genes such as *rpoC2*, *petB atpE*, *atpF* and *rpl22* showed lower similarity (average 80.2–83.5%) than other plastid genes in order (Table S1). Those genes with large sequence variations had evolutionary significance in inferring divergence times and branching patterns among early land plant lineages, while relatively less varied genes such as rRNA and PSII-associated genes were well conserved during the evolution of bryophytes.

### 2.3. Phylogenomic Analysis

Plastome information has provided an important resource for uncovering evolutionary relationships between various plant lineages [20,37]. The whole plastomes and protein-coding genes have been widely used for the reconstruction of phylogenetic relationships among different plant species [38]. The availability of completed *S. uncinata* plastome provided us with the sequence information to study the molecular evolution and phylogeny of *S. uncinata* with closely related species.

**Figure 3:**
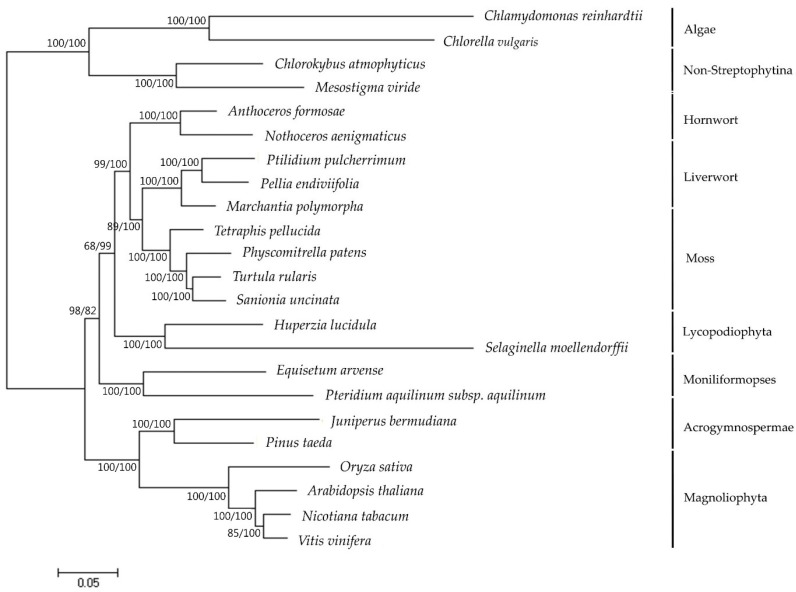
Phylogenetic tree reconstruction of 23 taxa using MEGA7 based on concatenated sequences of 40 protein-coding genes in the plastome. Maximum likelihood (ML) topology is shown with the bootstrap support values (MP/ML) given at nodes. Forty protein-coding sequences were extracted from annotated plastomes found in GenBank [21] (http://www.ncbi.nlm.nih.gov) (Table S2). The nucleotide sequences for each gene were translated into amino acids, aligned in MEGA7 and manually adjusted. Nucleotide sequences were aligned by constraining them to the amino acid sequence alignment. Individual gene alignments were then assembled into a single dataset.

Phylogenomic analysis of representatives from the bryophyte subfamily including *S. uncinata*, produced a single, well-supported tree using maximum parsimony (MP) (Figure 3). To do this, a set of 40 protein-coding genes in plastome analyzed in other species were concatenated and these concatenated sequences were used to infer the phylogenetic relationships of 23 taxa including *S. uncinata* using MEGA7. Phylogenetic analysis based on the multigene dataset revealed that mosses, liverwort and hornwort have been resolved as monophyletic in MP tree (Figure 3). Based on molecular phylogeny results, liverworts are placed in a basal position representing the earliest diverging lineage, while hornworts are the closest relatives of extant vascular plants, corroborating a previously reported branch order of “liverworts (mosses(hornworts(vascular plants)))” [37]. *S. uncinata* was most related to *T. ruralis* and formed a sister group with other moss species, which was supported by bootstrap values (100 for both ML and MP). In addition, it provides convincing support for many traditionally recognized genera and identifies higher level phylogenetic structure of mosses [39]. This result suggests that plastome information can effectively resolve phylogenetic positions and evolutionary relationships between different Bryophyte lineages.

The *T. ruralis* and *S. uncinata*, two mosses inhabiting extreme environments of polar and alpine regions, share common features in their plastomes–lack of *petN* and *rpoA* and presence of *trnP-GGG*–which is not the case for other two species of *P. patens* and *T. pellucid* (Table 4). Due to very scarce taxon sampling and the limitation of available plastome data, it is not clear whether the presence or absence of those genes is related to the resistance or adaptation to the extreme environments where they inhabit. To address this, synapomorphic characteristics developed during adaptation should be investigated together with genome evolution.

### 2.4. Loss of rpoA and petN in the S. uncinata Plastome

**Figure 4:**
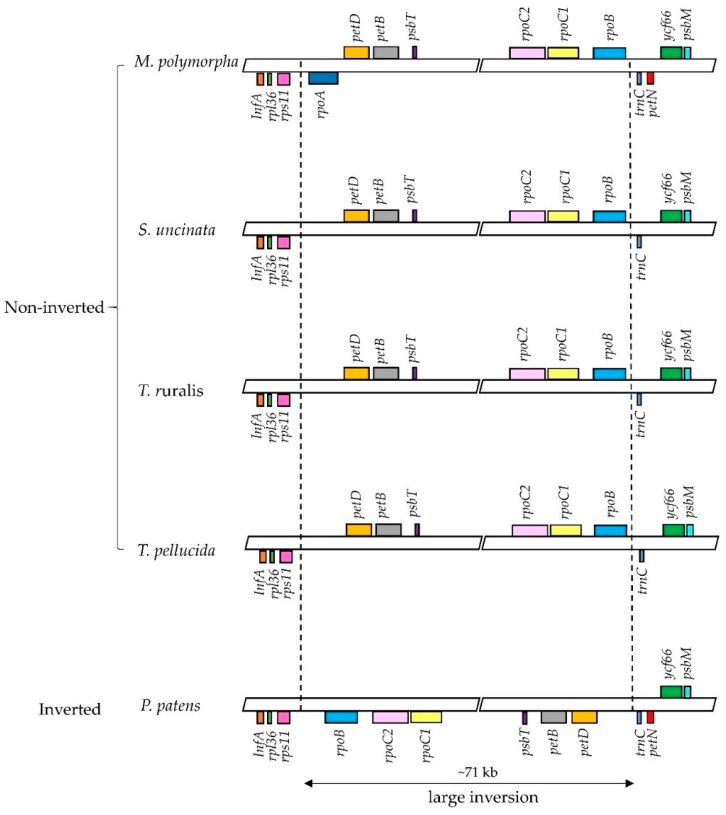
Comparison of the large inversion in the LSC region among six bryophytes plastomes. In comparative LSC region alignment of *rpoA*, *petN* coding regions from *M. polymorpha*, *S. uncinata*, *T. ruralis*, *T. pellucida* and *P. patens*. The inverted-arrangement of 71 kb fragment was only detected for *P. patens*.

Previous studies on bryophyte plastomes have shown that the overall structure and gene contents of plastomes are highly conserved among different bryophyte lineages [22,23,25,26]. The plastome of *S. uncinata* also showed a similar level of size and structure when compared to those of other three moss plastomes, *P. patens*, *T. ruralis* and *T. pellucida*. The plastome of *S. uncinata* did not show a large inversion of the ~71 kb fragment found in the LSC region of *P. patens* plastome, which follows the cases of *T. ruralis* and *T. pellucida*, suggesting that this large inversion is limited to the order *Funariales* in Bryophyte [22,40] (Figure 4).

While most mosses and plant groups are conserved in the contents of chloroplast genes, certain lineages undergo apparent and frequent gene loss (e.g., *rpoA* transfer from the chloroplast to the nucleus: [13,22,23]). Two genes encoding *α* subunit of RNA polymerase (*rpoA*) and encoding cytochrome b_6/f_ complex subunit VIII (*petN*) were absent in the *S. uncinata* plastome (Figure 4). As discussed in previous moss plastome studies, the most prominent and typical feature of the plastomes of moss is the absence of *rpoA*, which is thought to have disappeared together with *ycf5*, *cysA*, *cysT* after the divergence of the mosses from the hepatic bryophytes [22,41]. We could not identify *rpoA* with *cysT* and *cysA* in the *S. uncinata* plastome, which means that the *S. uncinata* plastome follows the typical characteristics of moss plastomes (Table 4, Figure 4). The loss of *petN* reported in other mosses, except in *P. patens* [22], was also observed in the *S. uncinata* plastome (Table 4, Figure 4). The presence of *trnP-GGG* also varied depending on species [13,23], the gene was present in the plastomes of *S. uncinata* and *T. ruralis*, while it was absent in the plastomes of *P. patens* and *T. pellucida* (Table 4).

### 2.5. Identification of Nuclear Genes Encoding rpoA and petN

Many cyanobacterial genes have been lost or transferred to the nuclear genomes during the transition from endosymbiont to organelle and the chloroplast genome might have been decreased in size [42,43]. Most gene loss occurred early in the endosymbiotic process; however, some losses have occurred in subsequent evolutionary processes [43]. The gene loss and transfer to the nucleus have been especially frequent, sporadic and temporary during the evolution of embryophytes and bryophytes [44]. Revealing why certain genes lose their functions is of great importance in the genome evolution of chloroplast, enabling the reconstruction of genomic events that occurred after the split of vascular plants and moss.

**Figure 5:**
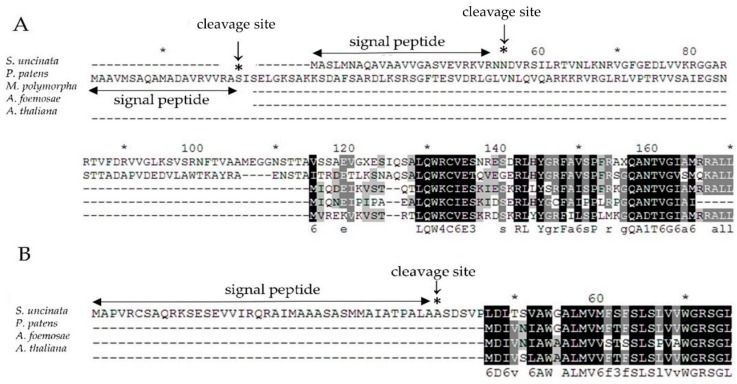
Amino acid alignment of (**A**) nuc-*rpoA* and (**B**) nuc-*petN* genes of *S. uncinata* with the nuc-*rpoA* or cp-*rpoA* and cp-*petN* genes from other green plants. Identical amino acid residues are boxed in black, other residues are printed in grey. Signal peptide sequences were predicted using SignalP [45] and shown as double arrow lines and the asterisk.

Therefore, to verify the chance of the nuclear relocation of the lost two chloroplast genes encoding the *α* subunit of RNA polymerase (*rpoA*) and cytochrome b_6/f_ complex subunit VIII (*petN*), we used blast to search for putative homologues against the draft sequences of the *S. uncinata* nuclear genome (unpublished results), as a result, we found one target sequence for each gene and then verified the sequences of PCR amplified fragments (nucleotide sequences are listed in Table S3), respectively, which might function as the replaced gene for *rpoA* or *petN* in *S. uncinata*. To check whether those nuclear genes would have a conserved function with chloroplastic genes, we performed a comparative alignment of nuc-*rpoA* (nuclear-*rpoA)* and nuc-*petN* (nuclear-*petN*) of *S. uncinata* with the homologous corresponding genes of *P. patens*, *M. polymorpha*, *A. formosae* and *A. thaliana* (Figure 5). Particularly, for *P. patens*, the nuc-*rpoA* sequence (*Pp* nuc-*rpoA*) was used in the alignment [20]. The nuc-*rpoA* sequence showed 41% identity with the nuc-*rpoA* sequence of *P. patens*, 76% of *M. polymorpha*, 66% of *A. formosae* and 67% of *A. thaliana* of the other cp-*rpoA* sequence from other compared species. The nuclear *petN* sequence showed 59–79% identified with the other chloroplastic *petN* sequence from other compared species *P. patens* (79%), *A. formosae* (59%) and *A. thaliana* (76%), respectively. When the sequences were scanned in the signalP 4.1 program [45] (SignalP 4.1 server; http://www.cbs.dtu.dk/services/Signal P), we could identify the signal peptide which targets chloroplast at the 5′ terminus of the nuc-*rpoA* gene of *S. uncinata* (Figure 5), suggesting that those genes would be localized in the chloroplast after being translated and functioned as other cp-*rpoA* or cp-*petN* genes in chloroplasts. This is very similar to the case of nuc-*rpoA* of *P. patens*, which has been shown to target the chloroplast organelle by the fluorescence-labelled protein method [22]. It would therefore be very interesting to prove that the proteins encoded by these nuclear genes actually constitute the chloroplastic-RNA polymerase complex or the Cyt b_6/__f_ complex in the moss protein expression system.

### 2.6. Prediction of RNA Editing Sites of Chloroplast Genes

Genetic information on DNA is sometimes altered at the transcript level by a process known as RNA editing, a sequence-specific post transcriptional modification resulting in the conversion, insertion, or deletion of nucleotides in a precursor RNA [46,47]. Such modifications are observed across organisms and have been reported with the discovery of C to U as well as U to C conversions in mitochondria and chloroplast in plants [48,49,50]. Since the first evidence of RNA editing in chloroplasts was found 24 years ago [51], RNA editing has been found in chloroplast transcripts from all major lineages of land plants [52]. In general, it is known that extensive RNA-editing occurs in hornworts and ferns when compared to seed plants [53]. The number of shared editing sites increases in closely related taxa, implying that RNA editing sites are evolutionarily conserved.

We predicted 72 editing sites in 22 protein coding genes by analyzing chloroplast coding sequences of *S. uncinata* using the PREP [54] and PREPACT 2.0-chloroplast program (http://www.prepact.de) [55] (Table S4). Most RNA editings are known to occur at the second codon position [56], with a frequency from 58.6% in hornwort to 68% in fern, 80% or 73.1% in *Cycad taitungensis* and *Pinus thunbergii*, respectively, 85.3%, 91.9% and 92% in *Arabidopsis thaliana*, *Nicotiana tabacum* and *Zea mays*, respectively, and up to 95.2% in *Oryza sativa* [57]. The RNA-editing of *S. uncinata* were mostly C-U editing events enriched at the second positions (64/72, 89%) as well, which allowed for the identification of the putative amino acid conversion by RNA editing (A-V, P-L, S-L, S-F, T-M and T-I).

## 3. Materials and Methods

### 3.1. Ethics Statement

Sample collection and field activities were carried out for scientific purposes in accordance with the Protocol on Environmental Protection to the Antarctic Treaty and approved by the Ministry of Foreign Affairs and Trade of the Republic of Korea (International Legal Affairs Division document No. 4029, approved on 18 December 2014). Sampling sites were not located within Antarctic Specially Protected Areas and no protected species were sampled in this study.

### 3.2. Plant Culture Conditions

*Sanionia uncinata* (Hedw.) Loeske plants growing under natural conditions were collected in the vicinity of the Korean King Sejong Antarctic Station (62°14′29′′ S; 58°44′18′′ W) on the Barton Peninsula of King George Island during the austral summer (mainly in January 2012) and then transferred to the lab and grown hydroponically in BCDAT solid media under a 16:8 h light:dark cycle at 15 °C.

### 3.3. Library Preparation and Sequencing

The total genomic DNA was extracted from tissues of plants using the DNeasy Plant Mini Kit (Qiagen). The quality of DNA was checked by Bioanalyzer 2100 (Aligent, Santa Clara, CA, USA). Library quantification was performed using the KAPA Library Quantification Kit (KAPA Biosystems, Boston, MA, USA). Paired-end cluster generation and sequencing were performed on the Illumina MiSeq, with each library being allocated to one lane of a flow cell. For the DNA library, TruSeq DNA sample preparation kits were used and sequenced in one lane of Illumina MiSeq 300 X 2 bp. The files containing the sequences and quality scores of reads were deposited in the NCBI Short Read Archive and the accession numbers are SRR6440975 genomic DNA-Seq (BioProject PRJNA428497).

### 3.4. Assembly and Annotation

After performing read preprocessing including adapter removal and quality filtering, the high-quality raw reads were aligned to 3 publicly available moss plastomes (*Physcomitrella patens* NC_005087, *Tortula ruralis* NC_012052 and *Tetraphis pellucida* NC_024291) downloaded from the NCBI organelle genome resources. The chloroplast reads were recovered from whole genome sequences by identifying them based on the alignment results and then de novo assembled using CLC Genomics Workbench V7.5 (CLC bio, Aarhus, Denmark). The assembled contigs were ordered based on their position in the reference plastome of *T. ruralis* because *T. ruralis* was identified as the top-hit species when BLAST searches were performed against the nr database. All gaps and junction regions between contigs and highly variable regions were validated by Sanger sequencing. The complete plastome was annotated using the program online software DOGMA [58] with default parameters, tRNAscan-SE [59] and BLAST similarity search tools from NCBI. OGDraw was used for map drawing [31]. The final *S. uncinata* plastome sequence has been submitted to GenBank (Accession: NC_025668).

### 3.5. Genome Alignment

The complete plastome sequences of 7 species were aligned using the mVISTA online suite [60]. A comparison of *Sanionia uncinata* (NC_025668) plastome structures with *Physcomiterella patens* (NC_005087), *Tortula ruralis* (NC_012052), *Tetraphis geniculate* (NC_024291), *Marchantia polymorpha* (NC_001319) and *Anthoceros formosae* (NC_004543), which are all in the bryophyte, was performed using the mVISTA program in Shuffle-LAGAN mode [60]. Default parameters were applied and the sequence annotation information of *S. uncinata* was used. Percentage identity between each plastome, all relative to that of *S. uncinata*, was subsequently visualized through an mVISTA plot. The mVISTA program only compared the sequence similarity by aligning the entire plastomes of all 6 taxa and the ~71 Kb inversion region of *P. patens* was reversed to match with other plastomes.

### 3.6. Phylogenetic Analysis

A set of 40 protein-coding genes, which have been analyzed in other species (accession numbers are listed in Table S2 and 40 protein-coding genes sequence of plastomes derived from 23 plants in Table S5), were used to infer the phylogenetic relationships among *S. uncinata*. Sequences were aligned using ClustalW. Phylogenetic analyses using maximum parsimony (MP) and maximum likelihood (ML) were performed, with MEGA7 [61]. For the MP analyses, the Subtree-Pruning-Regrafting algorithm was used with search level 1 in which the initial trees were obtained by the random addition of sequences (10 replicates). Sites with gaps or missing data were excluded from the analysis and statistical support was achieved through bootstrapping using 1000 replicates. For the ML analyses, the “Models” function of MEGA7 was used to find the best model for ML analysis. The Jones-Taylor-Thornton (JTT) + G + I + F model which was estimated to be the Best-Fit substitution model showing the lowest Bayesian Information Criterion, was employed in subsequent analyses. All positions containing gaps and missing data were eliminated. Bootstrap support was estimated with 1000 bootstrap replicates.

### 3.7. Prediction of RNA-Editing Sites

The RNA editing sites (C-to-U) in protein-coding genes were predicted by the online program Plant RNA Editing Prediction and Analysis Computer Tool (PREPACT 2.0) (http://www.prepact.de) [55] and Predictive RNA Editor for Plants (PREP) suite (http://prep.unl.edu/) [54] with a cutoff value of 0.8.

## 4. Conclusions

This study provided the whole sequence of the *S. uncinata* plastome, the dominant species of the Antarctic Peninsula, with information on the sequence and regulatory regions of chloroplast genes. We completed the *S. uncinata* plastome using a high-throughput sequencing method. The sequence and structure of *S. uncinata* were well conserved with the moss plastome sequences and was the most similar to the plastome sequence of *T. ruralis*. We confirmed that the two genes coding for *rpoA* and *petN* were lost in the *S. uncinata* plastome through comparative analysis of the plastome contents of the representative species of land plants and the sequence analysis results suggested the possibility of their relocation from plastome to nuclear genome. Our results also suggested that the possibility of post-transcriptional regulation of the chloroplast genes of *S. uncinata* by predicting the RNA-editing site. These results will contribute not only to the functional utilization of chloroplast genes but also to systematic phylogenetic analysis of land plants using whole plastome sequences.

## Figures and Tables

**Table 1:** Genes present in the *S. uncinata* plastome.

Gene Products	Genes
Photosystem I	*psaA*, *B*, *C*, *I*, *J*, *M*
Photosystem II	*psbA*, *B*, *C*, *D*, *E*, *F*, *H*, *I*, *J*, *K*, *L*, *M*, *N*, *T*, *Z*
Cytochrome b6/f	*petA*, *B ^a^*, *D ^a^*, *G*, *L*
ATP synthase	*atpA*, *B*, *E*, *F ^a^*, *H*, *I*
Translation factor	*infA*
Chlorophyll biosynthesis	*chlB*, *L*, *N*
Rubisco	*rbcL*
NADH oxidoreductase	*ndhA ^a^*, *B ^a^*, *C*, *D*, *E*, *F*, *G*, *H*, *I*, *J*, *K*
Large subunit ribosomal proteins	*rpl2 ^a^*, *14*, *16 ^a^*, *20*, *21*, *22*, *23*, *32*, *33*, *36*
Small subunit ribosomal proteins	*rps2*, *3*, *4*, *7*, *8*, *11*, *12 ^a^*^,*b*^, *14*, *15*, *18*, *19*
RNAP	*rpoB*, *C1 ^a^*, *C2*
Other proteins	*accD*, *cemA*, *clpP ^c^*, *matK*
Proteins of unknown function	*ycf1*, *2*, *3 ^c^*, *4*, *12*, *66 ^a^*
Ribosomal	*rrn4.5 ^d^*, *5 ^d^*, *16 ^d^*, *23 ^d^*
Transfer RNAs	*trnA(UGC) ^a^*^,*d*^, *C(GCA)*, *D(GUC)*, *E(UUC)*, *F(GAA)*, *G(UCC) ^a^*, *G(UCC)*, *H(GUG)*, *I(CAU)*, *I(GAU) ^a^*^,*d*^, *K(UUU) ^a^*, *L(CAA)*, *L(UAA) ^a^*, *L(UAG)*, *fM(CAU)*, *M(CAU)*, *N(GUU) ^d^*, *P(UGG)*, *P(GGG)*, *Q(UUG)*, *R(ACG) ^d^*, *R(CCG)*, *R(UCU)*, *S(GCU)*, *S(GGA)*, *S(UGA)*, *T(GGU)*, *T(UGU)*, *V(GAC) ^d^*, *V(UAC) ^a^*, *W(CCA)*, *Y(GUA)*

*^a^* Gene containing a single intron; *^b^* Gene divided into two independent transcription units; *^c^* Gene containing two introns; *^d^* Two gene copies in the IRs.

**Table 2:** The genes with introns in the *S. uncinata* plastome and the length of the exons and introns.

Gene	Location	Length (bp)				
		Exon I	Intron I	Exon II	Intron II	Exon III
*rps12*	LSC	114	-	270		
*ndhB*	LSC	729	629	780		
*ycf66*	LSC	106	591	320		
*rpoC1*	LSC	423	789	1614		
*atpF*	LSC	411	654	135		
*ycf3*	LSC	126	684	228	739	153
*clpP*	LSC	69	687	291	483	234
*rpl2*	LSC	396	637	438		
*ndhA*	SSC	556	731	551		
*trnK-UUU*	LSC	37	2272	42		
*trnL-UAA*	LSC	38	262	50		
*trnV-UAC*	LSC	37	542	37		
*trnI-GAU*	IR	42	769	35		
*trnA-UGC*	IR	38	763	35		

**Table 3:** The codon-anticodon recognition pattern and codon usage for *S. uncinata* plastome.

Amino Acid	Codon	No. *	tRNA	Amino Acid	Codon	No. *	tRNA
Phe	UUU	2862		Tyr	UAU	1492	
Phe	UUC	916	*trnF-GAA*	Tyr	UAC	578	*trnY-GUA*
Leu	UUG	688	*trnL-UAA*	Stop	UAA	1772	
Leu	UUA	1766	*trnL-CAA*	Stop	UAG	577	
Leu	CUG	252		His	CAU	550	
Leu	CUA	591	*trnL-UAG*	His	CAC	224	*trnH-GUG*
Leu	CUU	741		Gln	CAA	685	*trnQ-UUG*
Leu	CUC	305		Gln	CAG	273	
Ile	AUG	1582	*trnI-CAU*	Asn	AAU	1878	
Ile	AUU	1943		Asn	AAC	665	*trnN-GUU*
Ile	AUC	629	*trnI-GAU*	Lys	AAA	2942	*trnK-UUU*
Met	AUG	515	*trnfM-CAU*	Lys	AAG	768	
Val	GUG	244		Asp	GAU	619	
Val	GUA	588	*trnV-UAC*	Asp	GAC	210	*trnD-GUC*
Val	GUU	652		Glu	GAA	845	*trnE-UUC*
Val	GUC	237	*trnV-GAC*	Glu	GAG	286	
Ser	AGU	579		Cys	UGU	515	
Ser	AGC	470	*trnS-GCU*	Cys	UGC	362	*trnC-GCA*
Ser	UCG	272		Stop	UGA	657	
Ser	UCA	643	*trnS-UGA*	Trp	UGG	455	*trnW-CCA*
Pro	CCG	169		Arg	AGG	387	
Pro	CCA	429	*trnP-UGG*	Arg	AGA	732	*trnR-UCU*
Pro	CCU	423		Arg	CGG	164	*trnR-CCG*
Pro	CCC	230	*trnP-GGG*	Arg	CGA	322	
Thr	ACG	228		Arg	CGU	270	*trnR-ACG*
Thr	ACA	508	*trnT-UGU*	Arg	CGC	143	
Thr	ACU	561		Ser	UCU	735	
Thr	ACC	363	*trnT-GGU*	Ser	UCC	419	*trnS-GGA*
Ala	GCG	155		Gly	GGG	426	
Ala	GCA	367	*trnA-UGC*	Gly	GGA	465	*trnG-UCC*
Ala	GCU	413		Gly	GGU	653	
Ala	GCC	514		Gly	GGC	237	

* Numerals indicate the frequency of usage of each codon in 40,330 in codons in 82 potential protein-coding genes.

**Table 4:** Gene contents of plastomes from green alga, bryophytes and land plants.

	Plants *	Genome Size (bp)	*petN*	*rpoA*	*ccsA*	*cysA*	*cysT*	*ycf66*	*matK*	*rps15*	*trnP-GGG*
Alga	*Chlorella vulgaris*	150,613	-	+	+	+	+	-	-	-	-
Hornwort	*Anthoceros formosae*	161,162	+	+	+	+	+	-	Ψ	Ψ	+
Liverwort	*Marchantia polymorpha*	121,024	+	+	+	+	+	+	+	+	Ψ
Moss	*Tetraphis pellucida*	127,489	-	-	-	-	-	+	+	+	-
Moss	*Physcomitrella patens*	122,890	+	-	-	-	-	+	+	+	-
Moss	*Tortula rularis*	122,630	-	-	-	-	-	+	+	+	+
Moss	*Sanionia uncinata*	124,374	-	-	-	-	-	+	+	+	+
Lycopodiophyta	*Huperzia lucidula*	154,373	+	+	+	-	-	+	+	+	+
Moniliformopses	*Equisetum arvense*	133,309	+	+	+	-	-	+	+	+	+
Acrogymnospermae	*Pinus thunbergii*	116,635	+	+	+	-	-	-	+	+	+
Magnoliophyta	*Arabidopsis thaliana*	154,515	+	+	+	-	-	-	+	+	-

* The annotated plastomes are listed in Table S2*.* The presence ‘+’ or absence ‘-’ of each molecular character and pseudogenes are marked ‘Ψ’.

## Supplementary Materials

Supplementary materials can be found at http://www.mdpi.com/1422-0067/19/3/709/s1.

## Abbreviations

LSC	Large single copy
SSC	Small single copy
IR	Inverted repeat
Cp	Chloroplast
nuc	Nucleus
MP	Maximum parsimony
ML	Maximum likelihood
A	Adenine
T	Thymine
G	Guanine
C	cytosine
